# ACCEPTABILITY AND SATISFACTION WITH VIVO: PARTICIPANT FOCUS GROUPS AND SURVEY

**DOI:** 10.1093/geroni/igae098.1031

**Published:** 2024-12-31

**Authors:** Nathan Boucher, Elizabeth Kemp, Eric Levitan, Kathryn Porter Starr

**Affiliations:** Duke University, Durham, North Carolina, United States; Vivo, Atlanta, Georgia, United States; Vivo, Atlanta, Georgia, United States; Duke University School of Medicine, Hillsborough, North Carolina, United States

## Abstract

As part of the Vivo feasibility study, each participant (n= 22) completed a program satisfaction survey and participated in a focus group (four groups of 5-8 participants each). The satisfaction survey showed 1) 95% were satisfied with the program; 2) 86% indicated they would continue to do strength training exercises after the study; and 3) 77% indicated participation in Vivo made them more aware of the importance of strength training. To further examine acceptability and satisfaction with Vivo, our team conducted four focus groups focused on identifying key barriers and facilitators to participation in the virtual exercise sessions. Two coders, through individual coding and iterative team meetings with the larger team, used descriptive content analysis to code and identify common themes. Our team identified four themes: 1) self-reported positive outcomes (i.e., regained function after injury, variety of exercise kept interest); 2) positive exercise experience (i.e., manageable exercise with little equipment, felt inclusive, focus on safe exercise); 3) social connection with others (i.e., developed peer group, facilitate group discussion that helped bond group); and 4) importance of an individualized exercise program (i.e., modifiable depending on ability that day, individual feedback from trainers). Areas for improvement included: making rescheduling easier, optimizing trainer communication, and clarifying needed hardware. These findings support the acceptability and high satisfaction with virtual exercise programs for older adults with pre-diabetes.

